# Supplementary material for: Content-rich biological network constructed by mining PubMed abstracts
Source: BMC Bioinformatics. 2004 Oct 8;5:147. doi: 10.1186/1471-2105-5-147 (PMC528731; doi:10.1186/1471-2105-5-147)
Supplement: Additional File 2 — The original results of the above study (non-essential files are deleted to keep the file size under the limit set by BMC bioinformatics). [file 1471-2105-5-147-S2.bz2 › chilibotAdditionalFile2/dip10/467ID9195914E10495/html/IL8RB_CXCL1.html]

 


 **IL8RB** and **CXCL1** 
  
Found 79 abstracts in PubMed, retrieved 10.  
 

 What does Google say? 
 PDF only 
| .edu only 

---

**Interactive relationship** (e.g. stimulation, inhibition, etc)

**Neutral relationship**- The GRO  [ **CXCL1** ]  alpha receptor CXCR2  [ **IL8RB** ]  was also detected in tumor tissue, proposing interactions between eosinophils and the tumor.  Ref: 12734381 J Immunol, 2003
- Characterization of the molecular interactions of interleukin 8 CXCL8, growth related oncogen alpha  **CXCL1**  and a non peptide antagonist SB 225002 with the human CXCR2  [ **IL8RB** ] .  Ref: 12628493 Biochem Pharmacol, 2003
- Together these experiments support the notion that increased expression of KC  **CXCL1**  and MIP 2 CXCL2 3 and their interaction with CXCR2  [ **IL8RB** ]  are important in the pathogeneses of VILI.  Ref: 12464676 J Clin Invest, 2002
- These data indicate that HSPGs are involved in modulating  **CXCL1**  induced PAK1 activation and chemotaxis through regulating  **CXCL1**  binding activity to CXCR2  [ **IL8RB** ]  receptor.  Ref: 12549928 Biochemistry, 2003
- In addition, Gro  [ **CXCL1** ]  alpha was chemotactic for T cells expressing CXCR2  [ **IL8RB** ]  in vitro toward both recombinant protein and tumor derived chemokine.  Ref: 12427307 Hum Gene Ther, 2002
- ... several chemokines, including growth related oncogen alpha  **CXCL1**  and a non peptide antagonist SB 225002 are specific for CXCR2  [ **IL8RB** ] .  Ref: 12628493 Biochem Pharmacol, 2003
- CXCR2  [ **IL8RB** ]  binds IL 8 and other structurally related chemokines such as growth related oncogene GRO  [ **CXCL1** ]  a, GRObeta, GROgamma, neutrophil activating peptide 2 NAP 2 and epithelial cell derived neutrophil activating peptide 78 ENA 78.  Ref: 12365735 Fish Shellfish Immunol, 2002
- Interestingly we demonstrate, for the first time, that Gro  [ **CXCL1** ]  alpha was able to induce interferon gamma IFN gamma secretion from transduced T cells, thereby extending our knowledge of other potential functions of CXCR2  [ **IL8RB** ] .  Ref: 12427307 Hum Gene Ther, 2002
- CXCL10 inhibits  **CXCL1**  induced PAK1 activation and chemotaxis by interfering with appropriate binding of  **CXCL1**  to CXCR2  [ **IL8RB** ]  receptor.  Ref: 12549928 Biochemistry, 2003

**Non-interactive relationship** (e.g. studied together, co-existance, homology, etc.)

- In summary, we have shown that TII alveolar epithelial cells produce three of the major proinflammatory CXC chemokines, GRO  [ **CXCL1** ] , CINC 2alpha and MIP 2, and their cognate receptor CXCR2  [ **IL8RB** ] .  Ref: 12829448 Am J Respir Cell Mol Biol< MedlineTA>Am J Respir Cell Mol Biol, 2003
- Because the murine CXC chemokines KC  **CXCL1**  and MIP 2 CXCL2 3, via CXCR2  [ **IL8RB** ] , are potent neutrophil chemoattractants, we investigated their role in a murine model of VILI.  Ref: 12464676 J Clin Invest, 2002
- We show here that the  **CXCL1**  maximal binding to CXCR2  [ **IL8RB** ]  expressed on HEK293 and CHO K1 cells is dependent on the presence of cell surface HSPGs.  Ref: 12549928 Biochemistry, 2003
- However, T cells did not express the receptor for Gro  [ **CXCL1** ]  alpha, CXCR2  [ **IL8RB** ] , and therefore, T cells were transduced with a retroviral vector encoding CXCR2  [ **IL8RB** ] .  Ref: 12427307 Hum Gene Ther, 2002
- b for  **CXCL1** , the N terminus of CXCR2  [ **IL8RB** ]  is necessary but not sufficient for binding.  Ref: 12628493 Biochem Pharmacol, 2003
- Finally, the mechanism of binding of a non peptide antagonist on CXCR2  [ **IL8RB** ]  was investigated it occurred through epitopes a which were disseminated within the receptor, b which differed according to the use of CXCL8 or  **CXCL1**  as a competitor and c which did not necessarily overlap with agonist binding sites.  Ref: 12628493 Biochem Pharmacol, 2003
